# Experimental Testing and Constitutive Modelling of Pavement Materials

**DOI:** 10.3390/ma16114186

**Published:** 2023-06-05

**Authors:** Xueyan Liu, Yuqing Zhang, Zhanping You, Linbing Wang, Changhong Zhou

**Affiliations:** 1 Section of Pavement Engineering, Department of Engineering Structures, Delft University of Technology, 2628 CN Delft, The Netherlands; 2 Aston Institute of Materials Research (AIMR), Aston University, Birmingham B4 7ET, UK; y.zhang10@aston.ac.uk; 3 Department of Civil and Environmental Engineering, Michigan Technological University, 1400 Townsend Drive, Houghton, MI 49931, USA; zyou@mtu.edu; 4 School of Environmental, Civil, Agricultural and Mechanical Engineering, 1254 STEM Research Building II, University of Georgia, Athens, GA 30602, USA; linbing.wang@uga.edu; 5 School of Architecture and Transportation Engineering, Guilin University of Electronic Technology, Guilin 541004, China; czhou@guet.edu.cn

Pavement materials such as asphalt mixtures, granular aggregates and soils exhibit complex material properties and engineering performance under external loading and environmental conditions. For instance, the asphalt mixture shows highly nonlinear viscoelastic and viscoplastic properties at high temperatures, and it presents fatigue cracking damage and fracture properties at intermediate or low temperatures. The granular aggregate materials show an obvious anisotropic and stress-dependent resilient modulus. Their permanent deformation is fundamentally determined by stress levels, moisture and the number of load cycles. Constitutive models based on mechanics theories have been the kernel of performance prediction of pavement infrastructures and materials. They lay down a solid foundation for material selection, design and pavement structural evaluation, and maintenance decisions. Advances in mechanics modeling and the associated experimental testing for pavement infrastructures and construction materials are emerging constantly, such as nonlinear viscoelasticity, viscoplasticity, fracture and damage mechanics models. Meanwhile, various numerical modeling technologies are being developed and implemented to solve the multiscale and multi-physical equations and models for the pavement structures and materials. Examples include finite element, discrete element and micromechanics or molecular dynamics simulations at different dimensions and scales. These are being applied to both existing traditional pavement materials and novel or emerging materials such as recycled, modified or alternative materials. All the aforementioned advances have been leading to a large number of new studies and discoveries in the relevant areas.

This Special Issue provides a unique platform to collect and present these novel studies and new discoveries in the areas of mechanics, numerical modeling and the experimental testing of pavement infrastructures and materials. It includes the studies of various pavement materials such as asphalt concretes, granular materials, soils, recycled materials and additives. In addition, different testing and modeling technologies including discrete element modelling (DEM), computed tomography (CT) and molecular dynamics (MD) simulation are included.

A review paper summarizes the fatigue models of cement concrete pavements based on different testing scales [1]. Recommendations in terms of the data source, stress calculation method and regression analysis process were proposed for the improvement of current fatigue models for the cement concrete pavements.

Four papers focus on the characterization of different asphalt binders (e.g., polymer-modified, warm mix recycled and wax-modified binders) via experiments and molecular dynamics simulations [2,3,4,5]. The Dynamic Shear Rheometer (DSR)- and Bending Beam Rheometer (BBR)-based rheological tests are the mainstream methods to evaluate the high-, intermediate- and low-temperature performance of the asphalt binders. Fourier Transform Infrared (FTIR) Spectroscopy is widely used in terms of the chemical characterization of the functional groups in the asphalt binders. The Multiple Steep Creep and Recovery (MSCR) test and Linear Amplitude Sweep (LAS) test are used to investigate the effects of lignin and carbon fiber on the physical and mechanical properties’ changes in asphalt mastics [6].

Two papers focus on the evaluation of the asphalt pavement skid resistance. One investigated the effect of sand accumulation on the skid resistance of asphalt pavements using the British Pendulum Number (BPN) test on two types of asphalt mixtures [7]. Another one presented a finite element model of radial tire–asphalt pavement interaction to investigate the pavement skid resistance under partial tire aquaplane conditions [8]. The results showed that the vertical contact force between the tire and pavement is greatly reduced because of the partial aquaplane state.

Three papers utilize digital image processing (DIP) technology for either the performance test or meso-structure reconstruction of asphalt mixtures. The relationship between the rutting damage and the air void change was investigated via a 2D image technology [9]. An adaptive image processing method for CT images of asphalt mixtures was proposed to improve the accuracy of the meso-structure reconstruction of asphalt mixtures [10]. An improved procedure of the meso-structure reconstruction of asphalt mixtures considering the similarity of aggregate phase geometry was proposed, and the results indicated that the proposed approach can maintain the 3D spatial distribution features and contour characteristics of asphalt mixtures’ mesostructured [11]. One paper used the hexagonal close-packed (HCP) structure to establish the discrete model of asphalt mixtures for better simulating the shear failure [12]. The embedded sensor packaging of the rollpave pavements was optimized via experimental and numerical investigations [13]. This paper improved the compatibility of the embedded sensors and road materials in a prefabricated pavement structure, so the real-time in situ monitoring of the pavement response will be more accurate.

Six papers used laboratory tests and numerical simulations to assess the performance of different road materials and structures, including emulsified cold recycling asphalt mixtures, self-healing asphalt binder, reactive powder concrete and bridge deck pavement. The findings provide in-depth understandings in terms of various road materials key performance [14,15,16,17,18,19]. 

An efficient approach to obtain the parameters of the Prony series was proposed for the asphalt mixtures [20]. This method can simultaneously determine the retardation and relaxation spectra, which is more effective than the current approach. A fractional viscoelastic and damage constitutive relation of asphalt mixtures was proposed to characterize the three-stage creep process [21]. The model prediction results agreed well with the laboratory uniaxial compressive creep tests with different stress levels and temperatures. An improved mechanistic–empirical creep model considering the stress dependence and moisture sensitivity was proposed for the unsaturated soft and stabilized soils [22]. This developed model can predict the soil creep deformation under arbitrary water content and stress levels.
